# Right Ventricular Outflow Tract Mass in Antiphospholipid Syndrome

**DOI:** 10.1002/ccr3.70784

**Published:** 2025-08-08

**Authors:** Mohammad Sahebjam, Hamidreza Poorhosseini, Arezoo Haji Ali, Tayyebe Mohammad Gholizad, Saba Mohammadzadeh

**Affiliations:** ^1^ Department of Echocardiography, Tehran Heart Center, Cardiovascular Research Institute Tehran University of Medical Science Tehran Iran; ^2^ Department of Cardiology, Tehran Heart Center, Cardiovascular Research Institute Tehran University of Medical Science Tehran Iran

**Keywords:** antiphospholipid syndrome, intra cardiac thrombosis, right ventricular outflow tract thrombosis, transthoracic echocardiography

## Abstract

Antiphospholipid syndrome (APS) is well known for its association with arterial and venous thrombosis. Intracardiac thrombosis—more often involving the right atrium—is a serious manifestation of the disease. We describe an APS case with a thrombosis in the right ventricular outflow tract (RVOT), which underwent regression after aggressive anticoagulation.

## Graphical abstract

We represent a 49‐year‐old man with APS syndrome and history of multiple arterial and venous thrombosis, who presented with dyspnea. Transthoracic echocardiography revealed a mobile, round‐shaped, non‐homogeneous mass (14 × 8 mm), attached to RVOT, in favor of thrombosis. With aggressive anticoagulation the thrombosis regressed after about 1 month.

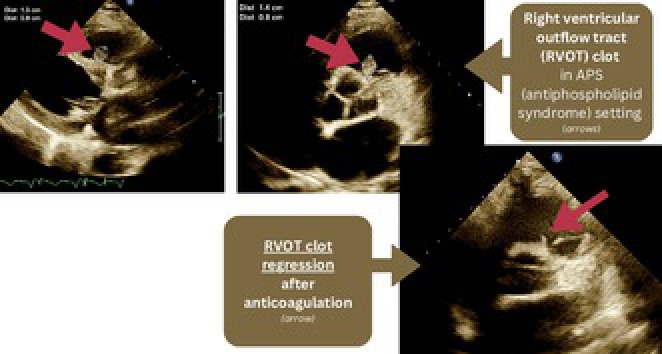

## 1 Introduction

Antiphospholipid syndrome (APS) is an autoimmune disorder defined by the presence of at least one laboratory and one clinical criterion. Laboratory criteria are persistent antiphospholipid antibodies (aPL)—including lupus anticoagulant (LAC), anticardiolipin (aCL), and anti‐β2 glycoprotein I (β2GPI) antibodies—detected in at least two tests conducted 12 weeks apart. The clinical criteria are recurrent arterial, venous, or small‐vessel thrombosis, pregnancy complications, or other nonthrombotic manifestations [1, 2].

Most cardiac complications of APS result from cardiovascular disease caused by accelerated atherosclerosis. Valvular involvement, ventricular dysfunction, intracardiac thrombi, and pulmonary hypertension could also complicate the APS course [3]. In a study of APS patients by transesophageal echocardiography (TEE), cardiac involvement (valvular thickening and/or regurgitation, vegetations and/or embolic sources) was found in 82% of primary APS patients. The most common abnormality among these was mitral valve thickening [4].

Thrombus formation in APS patients can occur in all cardiac chambers but more frequently in the right side of the heart (most commonly in right atrium [RA] followed by right ventricle [RV]), contrary to valve involvement, which is mostly in the left [4, 5, 6]. Intracardiac thrombi can cause pulmonary and systemic emboli, and in many cases, searching for the source of emboli leads to finding these thrombi [7]. Intracardiac thrombi in APS patients have been treated with aggressive anticoagulation therapy and/or surgical removal. However, substantial data supporting either approach is lacking [8].

Here, we present a case of RVOT thrombosis in the setting of primary APS in a middle‐aged man.

## 2 Case Presentation

### 2.1 History and Physical Examination

A 49‐year‐old male patient presented to our clinic with worsening dyspnea from 3 months ago. His past medical history was positive for polycythemia vera (PV), ischemic heart disease, primary antiphospholipid syndrome, deep vein thrombosis (DVT) and arterial thrombosis, as follows: He had repeated episodes of ST‐elevation myocardial infarction (STEMI) about 6 months ago, due to total thrombotic occlusion of coronary arteries and total in‐stent restenosis. At that time, in echocardiography, he had LVEF = 30% and aneurysmal LV apical segments with a large, fixed LV apical clot. He underwent coronary artery bypass graft (CABG), removal of LV clot, and repair of LV wall. Recurrent in‐stent restenosis had led to more laboratory investigations under rheumatologist consult, which revealed underlying primary antiphospholipid syndrome (positive Lupus anticoagulant (LAC), anticardiolipin (aCL) and anti β_2_‐glycoprotein І (β_2_ GPІ) antibodies). He also had an episode of acute deep vein thrombosis (DVT) of the left subclavian, axillary, brachial, basilic, cephalic, and ulnar veins, as well as left radial artery thrombosis during his last hospitalization.

The patient was on dual antithrombotic therapy (including clopidogrel and warfarin), with his last international normalized ratio (INR) = 2 (1 week before presentation). He had stopped taking ASA about 1 month ago.

Examination in this presentation revealed no cardiac murmur, clear breath sounds, and weak left radial pulse.

### 2.2 Investigations, Differential Diagnosis, and Treatment

**FIGURE 1:**
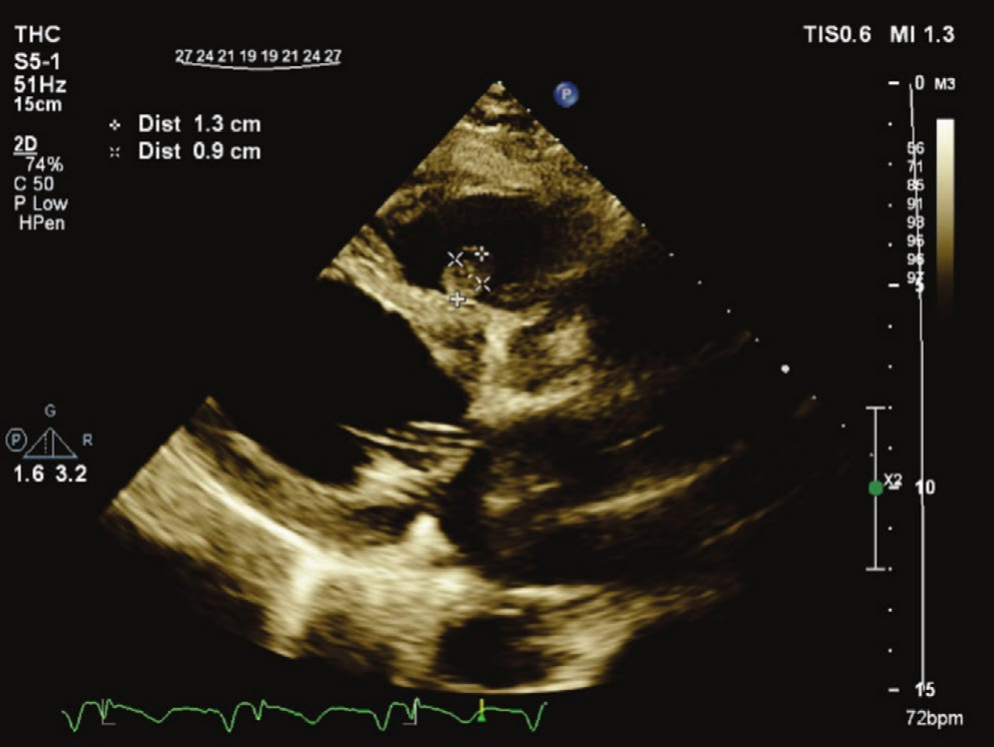
Transthoracic echocardiography in parasternal long axis, showing RVOT mass inserted close to pulmonary valve. RVOT, right ventricular outlet tract.

**FIGURE 2:**
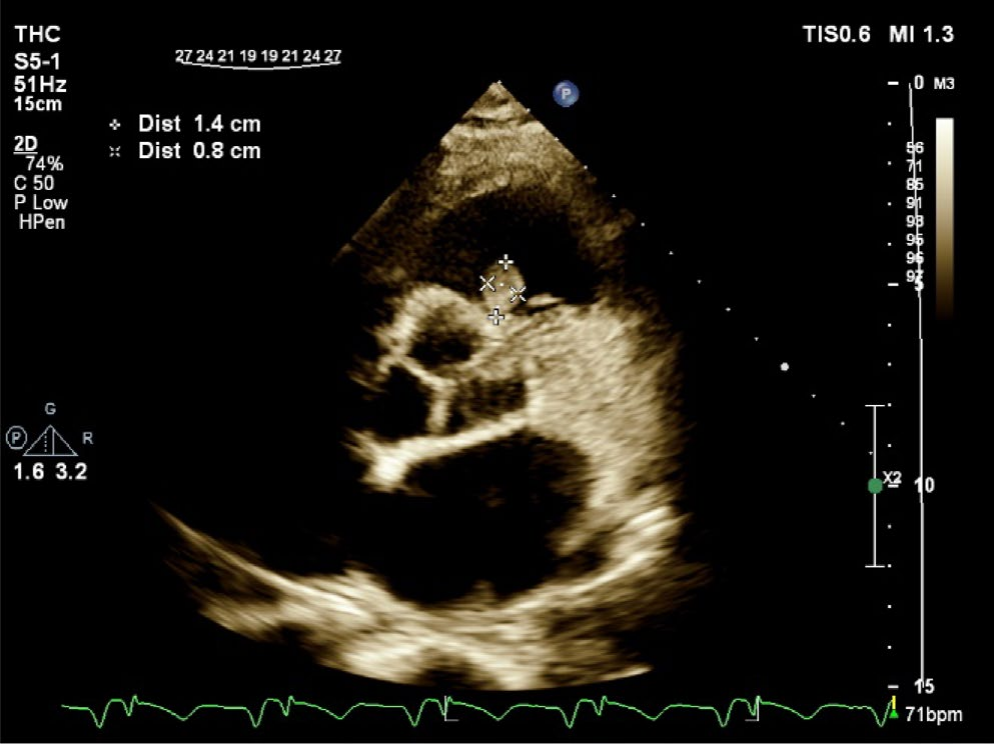
Transthoracic echocardiography in parasternal short axis, showing RVOT mass inserted close to pulmonary valve. RVOT, right ventricular outlet tract.

Transthoracic echocardiography showed a mobile, round‐shaped, non‐homogeneous mass (14 × 8 mm), attached to RVOT near the pulmonary valve as well as LVEF about 25%, large LV apical dead space, filled with clot (Figures 1 and 2; Videos 1 and 2).

**VIDEO 1:** Transthoracic echocardiography in parasternal long axis, showing mobile RVOT mass inserted close to pulmonary valve. RVOT, right ventricular outlet tract. Video content can be viewed at https://onlinelibrary.wiley.com/doi/10.1002/ccr3.70784.

**VIDEO 2:** Transthoracic echocardiography in parasternal short axis, showing mobile RVOT mass inserted close to pulmonary valve. RVOT, right ventricular outlet tract. Video content can be viewed at https://onlinelibrary.wiley.com/doi/10.1002/ccr3.70784.

The most probable diagnosis of the patient regarding his history of hypercoagulability state was thrombosis formation. The other differential diagnoses were cardiac tumor and endocarditis. The clinical setting of the patient, as he did not have fever or any sign of infection in laboratory data, was against endocarditis.

**FIGURE 3:**
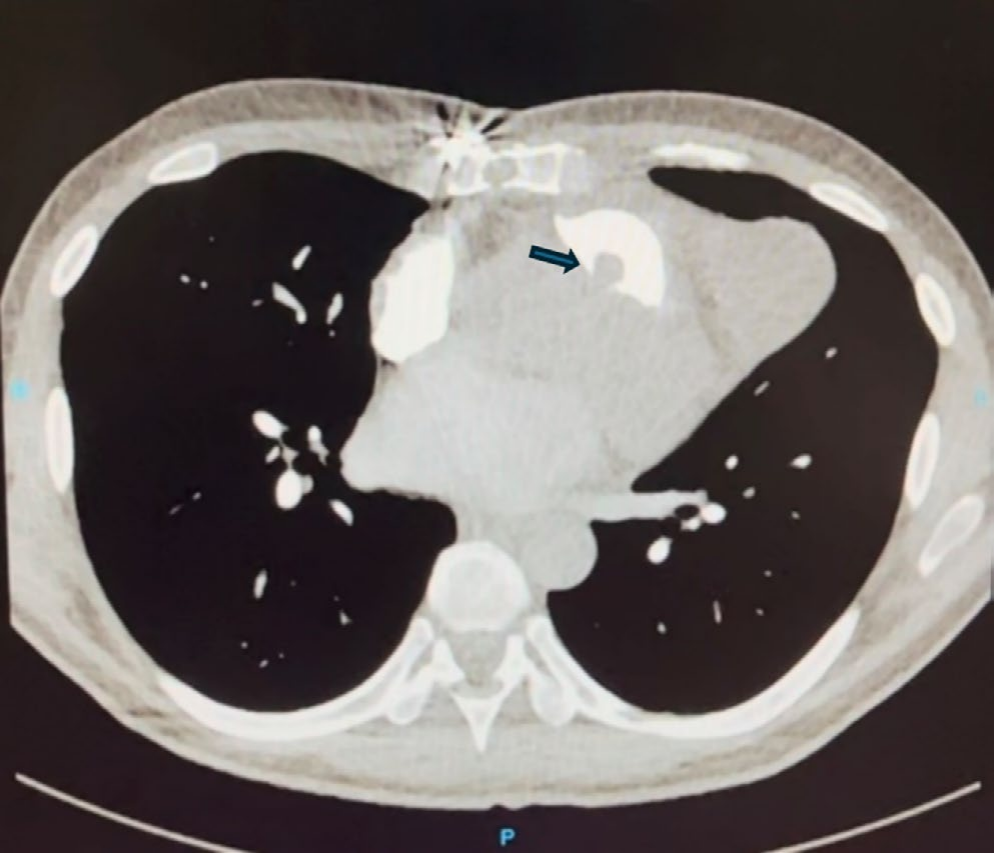
Computed tomography pulmonary angiogram showing a filling defect (15 × 10 mm) in RVOT suggestive of thrombosis (arrow). RVOT, right ventricular outlet tract.

Subsequent computed tomography pulmonary angiogram disclosed subsegmental pulmonary thromboembolism in the right lower lobe of the lung and a filling defect (15 × 10 mm) in RVOT, adherent to the subvalvular region, suggestive of thrombosis (Figure 3).

The patient was admitted to the cardiac care unit for further treatment. He received anticoagulation therapy with heparin and warfarin, along with antiplatelet and immunosuppressive drugs (including IVIG) under rheumatologist consult.

Laboratory investigation revealed high red blood cell (RBC) count (8.59 mil/μL) and otherwise normal routine laboratory tests (including normal white blood cell [WBC], platelet [PLT] count and normal erythrocyte sedimentation rate [ESR] and C‐reactive protein [CRP]) (Table 1).

**TABLE 1:** Showed features of APS, PV, and inflammation.

Parameter	Result	Reference range
White blood cell count (WBC)	9800 /μL	4000–100,000
Red blood cell count (RBC)	8.59 × 10^6^/μL	Male: 4.5–6
Hemoglobin (Hb)	14.7 g/dL	Male: 14–18
Platelet count (Plt)	286,000/μL	150,000–450,000
C‐reactive protein (CRP)	0.45 mg/dL	Normal < 0.5
Erythrocyte sedimentation rate (ESR)	1 mm/h	Normal < 20
Anti‐β2 glycoprotein I (IgM)	Positive	
Anti‐β2 glycoprotein I (IgG)	Positive	
Lupus anticoagulant	Positive	
Anticardiolipin antibodies	Positive	

Repeated follow‐up echocardiograms during hospitalization showed no significant change in RVOT mass's characteristics. Following discussion with the heart team regarding his high risk for re‐operative cardiac surgery, alleviation of symptoms, and stable mass size, the patient was discharged on warfarin with a target INR of 3.0–4.0.

### 2.3 Outcome and Follow‐Up

**FIGURE 4:**
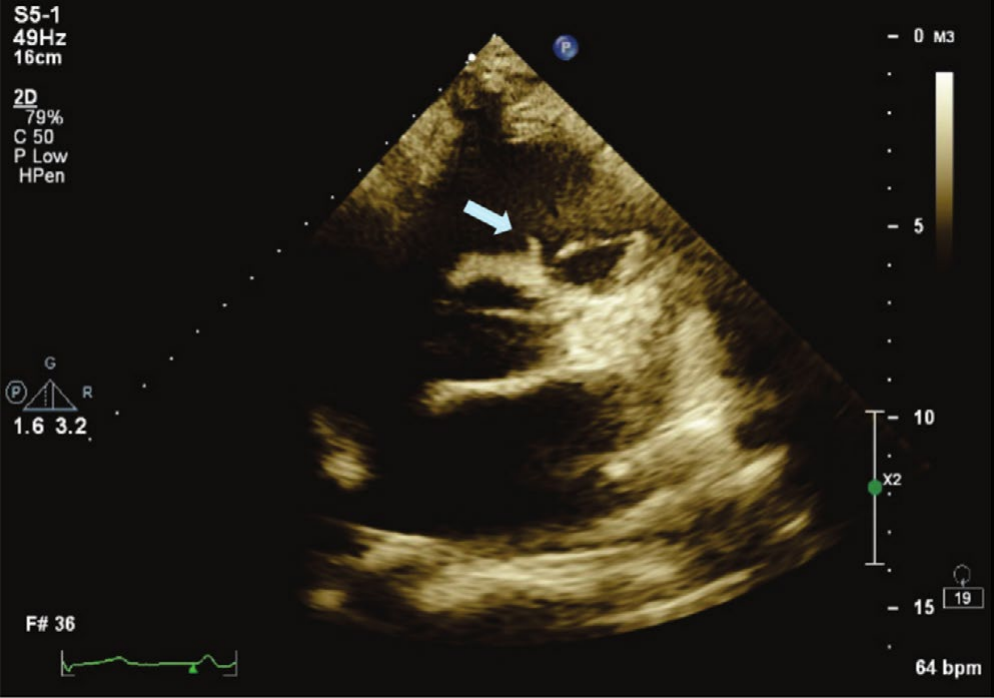
Transthoracic echocardiography in parasternal short axis, showing RVOT mass regression (3 × 7 mm) (arrow). RVOT, right ventricular outlet tract.

**FIGURE 5:**
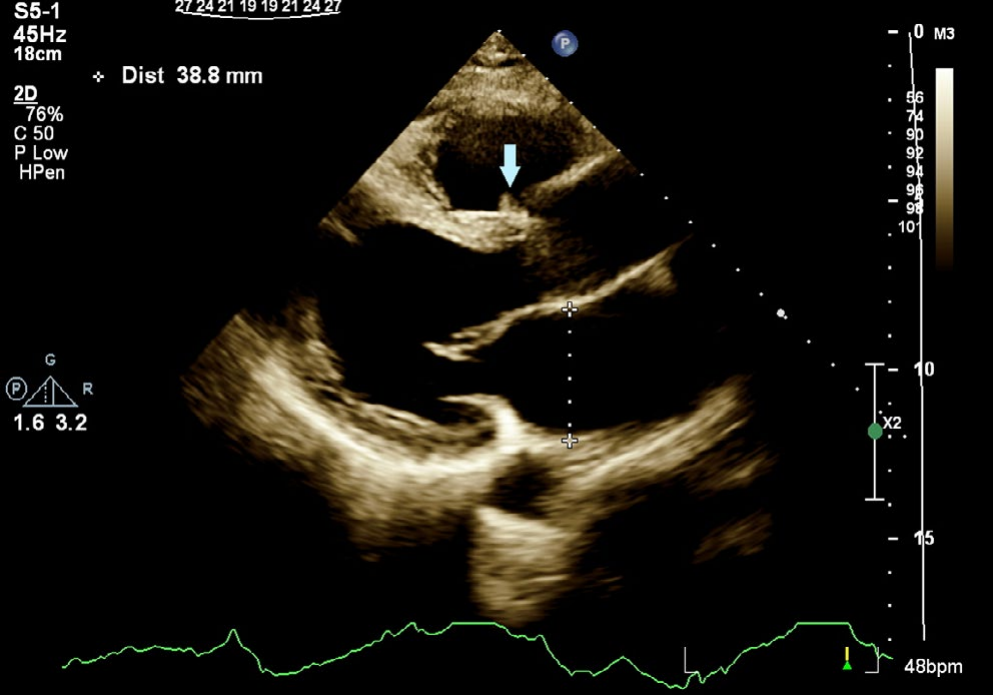
Transthoracic echocardiography in parasternal long axis, showing RVOT mass regression (arrow). RVOT, right ventricular outlet tract.

After about 1 month of anticoagulation therapy, follow‐up echocardiography showed significant regression in RVOT mass's size and only a small (3 × 7 mm) semimobile mass remained attached to RVOT (Figures 4 and 5). The patient denied any new complaints.

## 3 Discussion

APS is diagnosed by at least one episode of vascular thrombosis or recurrent pregnancy loss in patients with persistent antiphospholipid antibodies (aPL) performed at least 12 weeks apart [1, 2, 6]. Apart from atherosclerosis‐induced CVD, valvular involvement including thickening and vegetations is the most common cardiac involvement in APS [3, 9]. Other cardiac manifestations include coronary artery disease, myocardial dysfunction, pulmonary hypertension, and intracardiac thrombus [10]. These manifestations could be either secondary to immune‐mediated and/or thrombotic mechanisms [11]. 16% of primary APS patients had intracardiac thrombosis in a study by transesophageal echocardiography [12]. More commonly, thrombosis formation occurs on the surface of prosthetic or morphologically abnormal native heart valves than on structurally normal valve leaflets or on endo‐myocardium [13, 14, 15, 16, 17, 18]. In a review of literature published in 2016, among cardiac chambers, RA was the most frequently involved chamber in thrombosis formation in APS patients, and only 5 cases had RV thrombosis [6]. As far as we found, beyond the time of that review article in 2016, one more case of RV clot in the APS setting was reported in the literature, a case of catastrophic APS in a 12‐year‐old boy with right atrial thrombus attached to the tricuspid valve and extended into the right ventricular chamber. This patient had undergone surgical removal of the thrombus, but unfortunately expired [19]. Among all the reviewed articles, there was only one case of RVOT thrombosis in the APS setting, who was a middle‐aged Indian woman with RVOT obstruction secondary to an isolated organized thrombosis, managed by surgical excision of the clot [20]. Recently, a patient with APS was reported to have a calcified RVOT mass, which turned out to be a calcified amorphous tumor by histopathological examination [21].

In APS patients complicated with intracardiac thrombosis, aggressive maintenance anticoagulation therapy with heparin and warfarin (target INR = 3.0–4.0) is mandatory [12]. Although routine treatment for APS patients having thrombosis is giving oral vitamin K antagonist (VKA) to get a target INR of 2.0–3.0 [22], studies have shown recurrences of thrombosis formation mostly occur with actual INRs < 3.0 [23]. Direct oral anticoagulants (DOACs) can be used in the general population for the secondary prevention of thromboembolism, but are not routinely recommended for APS patients with thrombosis, especially in those having arterial thrombus or triple positive patients [24, 25].

The role of surgical excision of thrombosis is controversial. Randomized trials addressing the superiority of either approach in these patients are lacking [12].

In contrast to most of the previously presented intracardiac thrombus in the APS setting, which was managed surgically, the presented patient received aggressive anticoagulation regarding his high‐risk feature for re‐do surgery. Fortunately, the thrombosis regressed significantly after medical treatment.

Thrombotic mass is the most probable diagnosis for our patient, according to his past medical history and hypercoagulability state, as well as regression of the mass size after anticoagulation. However, infective endocarditis could be considered in the differential diagnosis of the mass; but the absence of fever and negative blood cultures argue against this possibility. A cardiac tumor should also be considered, although the observed regression in tumor size makes this diagnosis less likely.

In conclusion, although uncommon, intracardiac thrombosis, especially in the right chambers, should be considered and carefully searched for in patients with APS, particularly in the setting of PTE.

## Author Contributions

**Mohammad Sahebjam:** conceptualization, supervision, validation. **Saba Mohammadzadeh:** data curation, resources, writing – original draft, writing – review and editing. **Hamidreza Poorhosseini:** supervision. **Arezoo Haji Ali:** resources. **Tayyebe Mohammad Gholizad:** resources.

## Consent

Written informed consent was obtained from the patient for the publication of this case report, in accordance with the consent statement provided in the manuscript.

## Conflicts of Interest

The authors declare no conflicts of interest.

## Data Availability

The data that support the findings of this study are available on request from the corresponding author. The data are not publicly available due to privacy or ethical restrictions.
